# Supplementary material for: Discovery of Novel GMPS Inhibitors of Candidatus Liberibacter Asiaticus by Structure Based Design and Enzyme Kinetic
Source: Biology (Basel). 2021 Jun 28;10(7):594. doi: 10.3390/biology10070594 (PMC8301025; doi:10.3390/biology10070594)
Supplement: Supplementary file 1 [file biology-10-00594-s001.zip › GMPS supple material.pdf]

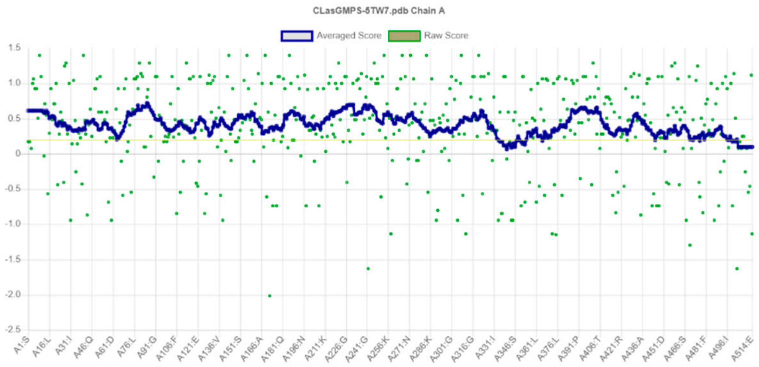

Figure S1. Verify 3D results of the refined CLas GMPS.

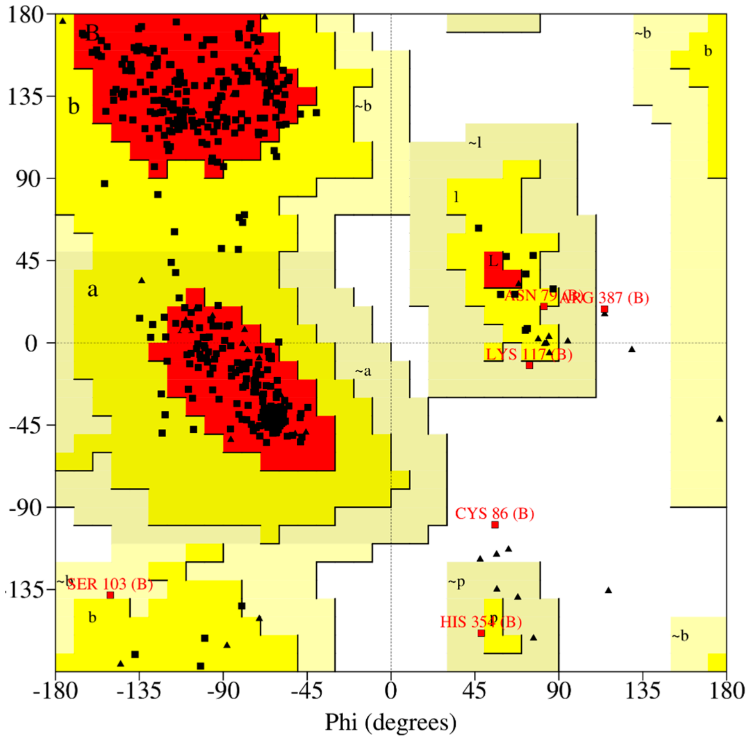

Figure S2. Ramachandran plot of CLas GMPS by PROCHECK.

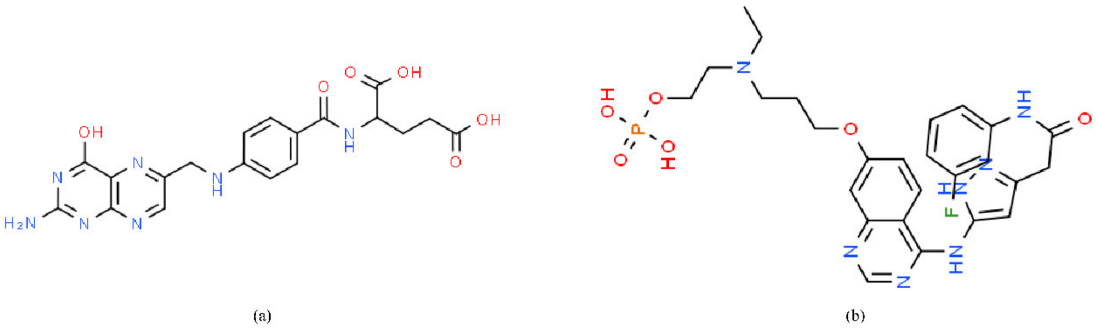

Figure S3. Structures of compounds selected from virtual screening and Enzyme Kinetic.

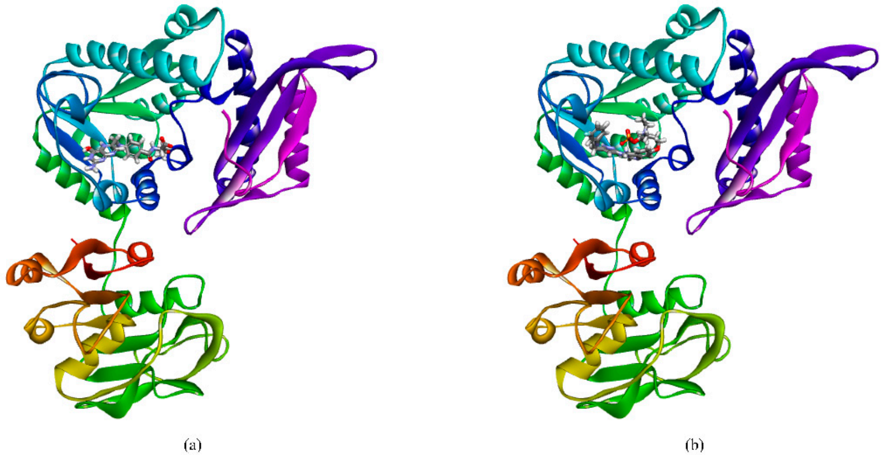

Figure S4. Schematic drawing of interactions between ligands and CLas GMPS. (a) Folic acid-CLas GMPS complex. (B) AZD1152- CLas GMPS complex.

Table S1. Compounds with higher LibDock.

Name	MW	LibScore
Compound1926	776.02	183.169
Compound2293	720.9	176.273
Compound5520	829.51	174.471
Compound1838	507.56	167.324
Compound2182	532.56	164.791
Compound1591	583.99	163.076
Compound5133	560.64	162.072
Compound5162	742.68	161.335
Compound2288	868.44	159.731
Compound2210	599.66	158.844
Compound4269	869.13	158.768
Compound2295	628.81	158.466
Compound174	589.71	155.534
Compound948	748.29	155.381
Compound1714	731.83	152.583
Compound4960	692.71	152.205
Compound1702	925.46	152.177
Compound5175	556.64	151.898
Compound2358	469.94	151.122
Compound1065	555.55	150.797
Compound4316	575.68	150.176
Compound3153	582.65	149.908
Compound6686	863.34	149.302
Compound4515	522.57	148.358
Compound4419	517.4	147.835
Compound1664	749.96	147.744
Compound3736	712.72	147.408

Compound2476	627.73	147.364
Compound1486	563.64	146.799
Compound4759	831.96	146.551
Compound1492	492.57	146.367
Compound3383	431.55	146.205
Compound4965	591.71	146.193
Compound1950	742.68	161.335
Compound1673	869.13	158.768
Compound5845	624.55	154.172
Compound955	925.46	152.177
Compound3971	863.34	149.302
Compound1430	712.72	147.408
Compound2101	664.43	147.381
Compound1123	627.73	147.364
Compound1841	831.96	146.551
Compound5481	441.4	145.107
Compound5609	811.67	144.256
Compound5868	584.66	144.022
Compound2265	735.05	143.103
Compound5741	640.2	141.749
Compound5608	594.52	140.529
Compound1334	587.54	191.661
Compound3170	776.02	183.169
Compound8511	636.79	182.643
Compound3997	720.9	176.273
Compound1226	551.64	174.129
Compound3975	813.43	171.437
Compound2704	507.56	167.324
Compound8477	669.79	166.047
Compound7726	571.57	165.201
Compound2503	521.67	165.127
Compound3752	532.56	164.791
Compound4073	529.53	163.076
Compound6234	494.59	162.396
Compound5313	576.62	162.366
Compound4979	634.73	162.348
Compound8081	560.64	162.072
Compound1446	666.77	160.757
Compound5292	630.66	159.817
Compound4036	591.48	159.783
Compound3982	868.44	159.731
Compound3799	599.66	158.844
Compound2351	587.67	158.547
Compound1249	607.69	158.518

Compound3999	628.81	158.466
Compound6966	430.55	158.195
Compound8826	581.37	157.774
Compound448	589.71	155.534
Compound1276	748.29	155.381
Compound4142	523.67	155.248
Compound6008	491.49	155.022
Compound8101	624.17	154.492
Compound2456	437.54	154.38
Compound10000	624.55	154.172
Compound8663	569.58	153.954
Compound531	695.73	153.296
Compound741	479.96	153.135
Compound2534	731.83	152.583
Compound5505	635.93	152.541
Compound2397	517.4	152.426
Compound2639	508.55	152.362
Compound7793	692.71	152.205
Compound2512	925.46	152.177
Compound8269	556.64	151.898
Compound4966	660.52	151.868
Compound7203	469.58	151.629
Compound2240	489.64	151.464
Compound4278	469.94	151.122
Compound2214	490.55	151.097
Compound4010	646.73	150.982
Compound3916	527	150.869
Compound1400	555.55	150.797
Compound8831	504.63	150.778
Compound5859	633.69	150.648
Compound7963	382.38	150.557
Compound981	567.01	150.368
Compound6714	575.68	150.176
Compound532	652.71	149.986
Compound5795	582.65	149.908
Compound8263	524.68	149.753
Compound529	668.66	149.512
Compound4576	618.51	148.47
Compound6853	522.57	148.358
Compound1186	397.48	147.844
Compound6782	517.4	147.835
Compound2379	749.96	147.744
Compound3035	394.47	147.63
Compound1516	705.85	147.622

Compound8524	463.46	147.477
Compound6429	712.72	147.408
Compound4452	627.73	147.364
Compound1472	563.64	146.799
