# Supplementary figures and images for: Discovery of Novel GMPS Inhibitors of Candidatus Liberibacter Asiaticus by Structure Based Design and Enzyme Kinetic
Source: Biology (Basel). 2021 Jun 28;10(7):594. doi: 10.3390/biology10070594 (PMC8301025; doi:10.3390/biology10070594)

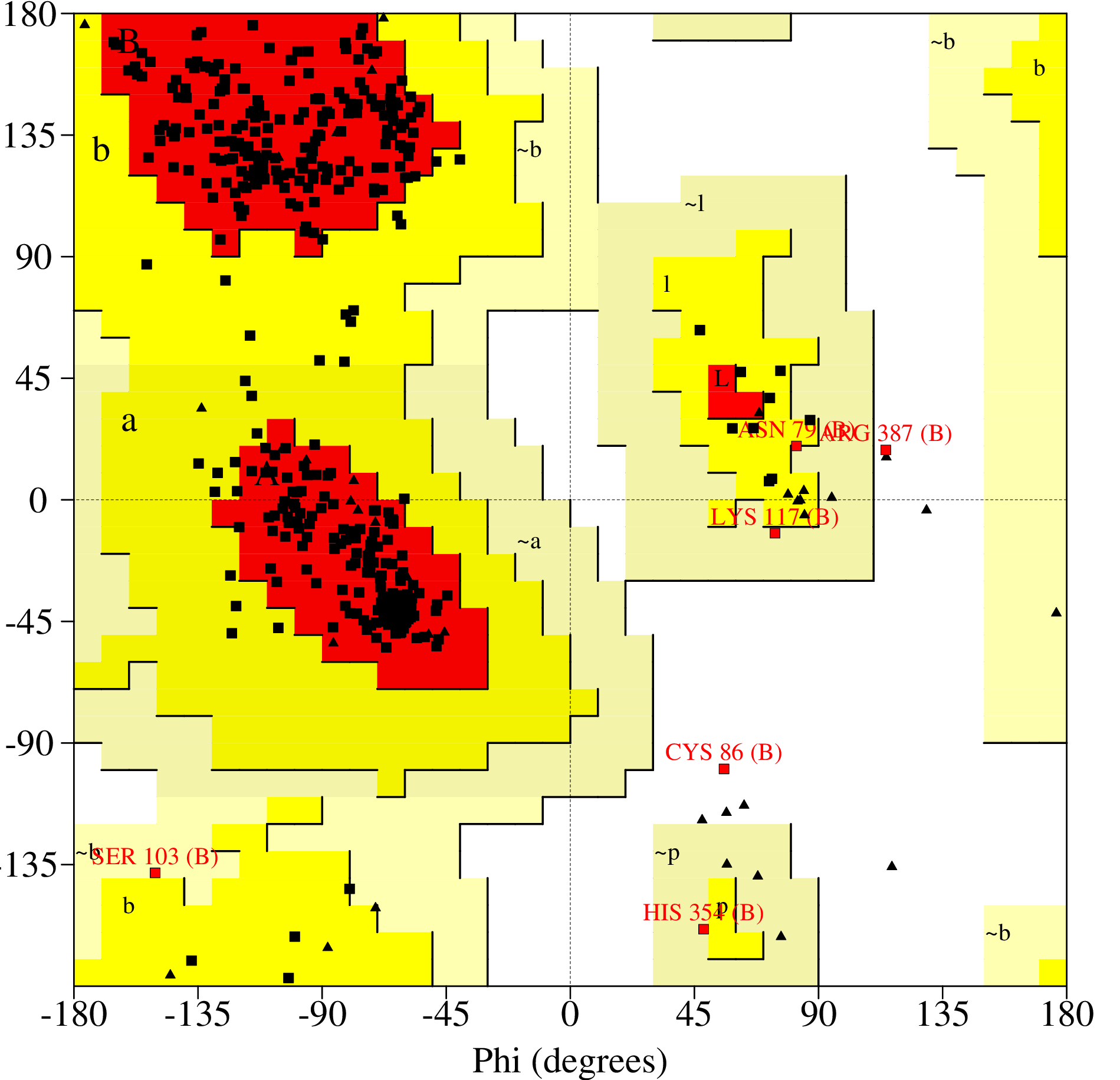

Supplement: Supplementary file 1 [file biology-10-00594-s001.zip › Figure S2.png]
